# A DNA Methylation-Based Test for Breast Cancer Detection in Circulating Cell-Free DNA

**DOI:** 10.3390/jcm7110420

**Published:** 2018-11-07

**Authors:** Sofia Salta, Sandra P. Nunes, Mário Fontes-Sousa, Paula Lopes, Micaela Freitas, Margarida Caldas, Luís Antunes, Fernando Castro, Pedro Antunes, Susana Palma de Sousa, Rui Henrique, Carmen Jerónimo

**Affiliations:** 1 Cancer Biology & Epigenetics Group—Research Center, Portuguese Oncology Institute of Porto (CI-IPOP), 4200-072 Porto, Portugal; sofia.salta@ipoporto.min-saude.pt (S.S.); sandra22nunes@hotmail.com (S.P.N.); ana.ambrosio@ipoporto.min-saude.pt (P.L.); micaelafariafreitas@gmail.com (M.F.); margaridabcaldas@yahoo.co.uk (M.C.); rmhenrique@icbas.up.pt (R.H.); 2 Master in Oncology, Institute of Biomedical Sciences Abel Salazar—University of Porto (ICBAS-UP), 4050-313 Porto, Portugal; 3 Breast Cancer Clinic and Department of Medical Oncology, Portuguese Oncology Institute of Porto, 4200-072 Porto, Portugal; mfontes.sousa@ipoporto.min-saude.pt (M.F.-S.); susana.sousa@ipoporto.min-saude.pt (S.P.d.S.); 4 Department of Pathology, Portuguese Oncology Institute of Porto, 4200-072 Porto, Portugal; 5 Department of Epidemiology, Portuguese Oncology Institute of Porto, 4200-072 Porto, Portugal; luis.antunes@ipoporto.min-saude.pt; 6 Breast Cancer Clinic and Department of Surgical Oncology, Portuguese Oncology Institute of Porto, 4200-072 Porto, Portugal; fcastro@ipoporto.min-saude.pt (F.C.); pedrobiniantunes@gmail.com (P.A.); 7 Department of Pathology and Molecular Immunology, Institute of Biomedical Sciences Abel Salazar—University of Porto (ICBAS-UP), 4050-313 Porto, Portugal

**Keywords:** breast cancer, DNA methylation, epigenetic biomarker, Cell-free DNA, liquid biopsy, diagnosis, prognosis

## Abstract

Background: Breast cancer (BrC) is the most frequent neoplasm in women. New biomarkers, including aberrant DNA methylation, may improve BrC management. Herein, we evaluated the detection and prognostic performance of seven genes’ promoter methylation (*APC*, *BRCA1*, *CCND2*, *FOXA1*, *PSAT1*, *RASSF1A* and *SCGB3A1*). Methods: Methylation levels were assessed in primary BrC tissues by quantitative methylation-specific polymerase chain reaction (QMSP) and in circulating cell-free DNA (ccfDNA) by multiplex QMSP from two independent cohorts of patients (Cohort #1, *n* = 137; and Cohort #2, *n* = 44). Receiver operating characteristic (ROC) curves were constructed, and log-rank test and Cox regression were performed to assess the prognostic value of genes’ methylation levels. Results: The gene-panel *APC*, *FOXA1*, *RASSF1A*, *SCGB3A1* discriminated normal from cancerous tissue with high accuracy (95.55%). In multivariable analysis, high *PSAT1*-methylation levels [>percentile 75 (P75)] associated with longer disease-free survival, whereas higher *FOXA1*-methylation levels (>P75) associated with shorter disease-specific survival. The best performing panel in ccfDNA (*APC*, *FOXA1* and *RASSF1A*) disclosed a sensitivity, specificity and accuracy over 70%. Conclusions: This approach enables BrC accurate diagnosis and prognostic stratification in tissue samples, and allows for early detection in liquid biopsies, thus suggesting a putative value for patient management.

## 1. Introduction

Breast cancer (BrC) is the most common and lethal cancer in women worldwide, corresponding to 25% of all cancers in females [1]. Implementation of mammography-based BrC screening increase the proportion of cancers detected at an early-stage, contributing to a decrease in BrC-related mortality [2]. Nevertheless, this screening strategy is hampered by frequent false positive results, leading to overdiagnosis. Furthermore, its usefulness in women with dense breast tissue remains controversial [3,4]. Although grade, stage, histological, and molecular subtype are currently used to risk-stratify BrC patients, divergent outcomes and therapeutic responses are common [5]. Furthermore, currently used prognostic and predictive biomarkers, such as hormone receptor or *Erb-b2 receptor tyrosine kinase 2* (ERBB2) status have a limited power to predict recurrence and therapeutic response [6]. Hence, despite all improvements in early detection, patients’ stratification and treatment, BrC remains the foremost cause of cancer-related mortality among women, mostly due to disease recurrence and/or metastasis development [1]. In recent years, several biomarkers for early diagnosis have been proposed. Yet, despite their less invasive nature [7,8,9], improved tumor characterization [10,11,12,13] or better patient stratification [9,14] have been proposed, but with limited success.

Because aberrant DNA methylation is considered a cancer-associated event, characterization of tumor-specific methylome has become the focus of multiple studies [15]. Interestingly, aberrant promoter methylation of several tumor suppressor genes was found in BrC precursor lesions, indicating that DNA methylation is an early event in breast carcinogenesis [16,17,18,19]. Moreover, DNA methylation has been proposed as a valuable cancer detection and prognosis biomarker owing to its link with tissue-specific gene silencing [9,20,21,22,23]. Tumor-specific DNA methylation may also be detected in circulating cell-free DNA (ccfDNA) from liquid biopsies [24], and its potential for early cancer detection was already reported [23,25,26,27], representing a minimal-invasive test [28]. Herein, we aimed to define a DNA methylation-based test to improve or complement early detection strategies and to enable better BrC patients’ prognostic stratification. Thus, methylation levels of seven gene promoters [*Adenomatosis polyposis coli (APC)*, *BRCA1*, *DNA repair associated (BRCA1)*, *Cyclin D2 (CCND2)*, *Fork-head box A1 (FOXA1)*, *Phosphoserine Aminotransferase 1* (*PSAT1)*, *Ras association domain family 1 isoform A (RASSF1A)* and *Secretoglobin family 3A member 1 (SCGB3A1*)] previously reported as dysregulated in BrC and conveying diagnostic and/or prognostic information [7,8,9,14,29] were firstly assessed in tissue for confirmation of cancer-specificity and prognostic significance. Then, the best performing gene panel was tested in plasma ccfDNA to determine its BrC detection performance.

## 2. Experimental Section

### 2.1. Patients and Samples Collection

Two independent cohorts of BrC patients were included in this study. Cohort #1 comprised 137 patients, primarily submitted to surgery, from 1996 to 2001, at the Portuguese Oncology Institute of Porto (IPO Porto), with frozen tissue available. For control purposes, normal breast tissue (NBr) was collected from reduction mammoplasty of contralateral breast of BrC in patients without BrC hereditary syndrome. After surgical resection and examination, samples were immediately frozen at −80 °C. Relevant clinical and pathological data was retrieved from the patients’ clinical charts. Five μm frozen sections were cut and stained by hematoxilin-eosin for histological evaluation by an experienced pathologist.

Cohort #2 was composed of 44 BrC patients, primarily diagnosed between 2015 and 2017 at IPO Porto, which voluntarily provided blood samples prior any treatment. For control purposes, blood samples were also obtained from 39 asymptomatic controls (AC). The blood samples were collected in two EDTA tubes and centrifuged at 2000 rpm for 10 min at 4 °C for plasma separation. Plasma was immediately frozen at −80 °C until further use. Relevant clinical data was collected from clinical records.

This study was approved by the institutional review board (Comissão de Ética para a Saúde—CES 120/2015) of IPO Porto, Portugal. All patients and controls enrolled had signed an informed consent.

### 2.2. Immunohistochemistry 

Immunohistochemistry (IHC) allowed for identification of BrC molecular subtype of each case in Cohort #1, using corresponding formalin-fixed paraffin-embedded tissue. Commercially available antibodies for Estrogen Receptor (ER) (Clone 6F11, mouse, Leica, Newcastle, UK), Progesterone Receptor (PR) (Clone 16, mouse, Leica, Newcastle, UK), ERBB2 (Clone 4B5, rabbit, Roche, Tucson, AZ, USA) and Ki67 (Clone MIB-1, mouse, Dako, Glostrup, Denmark) were used. IHC was carried out in BenchMark ULTRA (Ventana, Roche, Tucson, AZ, USA) using ultraView Universal DAB Detection Kit (Ventana, Roche, Tucson, AZ, USA) according to manufacturer’s instructions. 

IHC staining was evaluated by an experienced pathologist according to College of American Pathologists’ recommendations. Each case was categorized according to European Society for Medical Oncology (ESMO) guidelines [6]. Cutoff values were set for Ki67 (high proliferative rate if ≥15% positive cells) and PR (high expression if ≥25% positive cells).

### 2.3. DNA Extraction

Genomic DNA was extracted from tumor and normal tissues by the phenol–chlorophorm method at pH 8, as previously described [30]. Samples were first submitted to overnight digestion in a bath at 55°C, using buffer solution SE (75 mM NaCl; 25 mM EDTA), SDS 10% and proteinase K, 20 mg/mL (Sigma-Aldrich^®^, Schnelldorf, Germany). After digestion, extraction was performed with phenol/chloroform (Sigma-Aldrich^®^, Schnelldorf, Germany, Merck, Darmstadt, Germany) followed by precipitation with 100% ethanol.

CcfDNA was extracted from 2 mL of plasma using QIAamp MinElute ccfDNA (Qiagen, Hilden, Germany), according to manufacturers’ recommendations. The ccfDNA was eluted in 20 µL of ultra-clean water (Qiagen, Hilden, Germany).

### 2.4. Bisulfite Treatment and Whole Genome Amplification (WGA)

Bisulfite conversion was performed using the EZ DNA Methylation-Gold Kit (Zymo Research, Orange, CA, USA), according to manufacturer’s instructions. One µg of DNA obtained from fresh frozen sections was used. Modified DNA was eluted with 60 μL of sterile distilled water. In plasma samples, 20 μL of ccfDNA was used for bisulfite modification. Modified ccfDNA was eluted in 10 μL of sterile distilled water. For control purposes, 1 μg of CpGenome™ Universal Methylated DNA (Millipore, Temecula, CA, USA) was also modified, according to the method described above and eluted in 20 μL of M-elution buffer. All samples were stored at −80 °C until further use. Whole-genome amplification of sodium bisulfite modified ccfDNA was carried out using the EpiTect Whole Bisulfitome Kit (Qiagen, Hilden, Germany) according to manufacturer’s recommendations. The amplified ccfDNA final volume was 65 μL.

### 2.5. Quantitative Methylation-Specific Polymerase Chain Reaction (QMSP) 

Modified DNA was used as template for QMSP. Overall, seven gene promoters (*APC*, *BRCA1*, *CCND2*, *FOXA1*, *PSAT1*, *RASSF1A* and *SCGB3A1*) were assessed in BrC tissue samples. The primers used specifically amplify methylated bisulfite converted complementary sequences and are listed in Supplementary File 1 (Table S1). *β-actin* (*ACTβ*) was used as reference gene to normalize for DNA input in each sample [9]. Reactions were performed in 96-well plates using Applied Biosystems 7500 RealTime System (Applied Biosystems, Perkin Elmer, CA, USA) using 2 μL of modified DNA and 5 μL of 2X KAPA SYBR FAST qPCR Master Mix (Kapa Biosystems, Wilmington, MA, USA). All the samples were run in triplicates and the melting curves were obtained for each case/gene. Owing to the limited amount of ccfDNA plasma samples, three gene promoters were selected (*APC*, *FOXA1*, *RASSF1A*) in addition to the reference gene (*ACTβ*) for assessment of methylation using multiplex QMSP with TaqMan probes having different fluorescent reporters and Xpert Fast Probe (GRISP, Porto, Portugal), whereas *SCGB3A1* methylation levels were assessed in a separated QMSP reaction (Supplementary File 1—Table S2). 

Modified CpGenome™ Universal Methylated DNA was used in each plate to generate a standard curve, allowing for quantification, as well as to ascertain polymerase chain reaction (PCR) efficiency. All plates disclosed efficiency values between 90–100%. For each gene, relative methylation levels in each sample were determined by the ratio of the mean quantity obtained by QMSP analysis for each gene and the respective value of the internal reference gene (*ACTβ*), multiplied by 1000 for easier tabulation (methylation level = target gene/reference gene × 1000).

### 2.6. Statistical Analysis

The frequency, median and interquartile range of promoter methylation levels of normal tissue/control samples and plasma samples were determined. Non-parametric tests were performed to determine statistical significance in all the comparisons made. Specifically, Kruskall-Wallis test was used for comparisons between three or more groups, whereas Mann-Whitney U test was used for comparisons between two groups. 

For each gene, receiving operating characteristic (ROC) curves were built to assess respective performance as tumor biomarker. Moreover, specificity, sensitivity, positive predictive value (PPV), negative predictive value (NPV), accuracy were determined. For this purpose, the cut-off established was the highest value obtained by the ROC curve analysis [sensitivity + (1 − specificity)]. To categorize samples as methylated or unmethylated, a cut-off value was chosen based on Youden’s J index obtained by the ROC curve analysis for each gene [31,32]. For combination of markers, the cases were considered positive if at least one of the individual markers was positive. Logistic regression models were built in order to evaluate the potential of confounding factors as age in our BrC detection model.

Spearman nonparametric correlation test was used to assess the association of methylation levels and age. Disease-specific survival curves and disease-free survival curves (Kaplan–Meier with log rank test) were computed for standard clinicopathological variables and for categorized methylation status. A Cox-regression model comprising all significant variables (multivariable model) was computed to assess the relative contribution of each variable to the follow-up status. 

Two-tailed *p*-values were derived from statistical tests, using a computer assisted program (SPSS Version 20.0, Chicago, IL, USA), and results were considered statistically significant at *p* < 0.05, with Bonferroni’s correction for multiple tests, when applicable. Graphics were assembled using GraphPad 6 Prism (GraphPad Software, La Jolla, CA, USA).

## 3. Results

### 3.1. Clinical and Pathological Data of Tissue Cohort

Relevant clinical and pathological data are presented in Supplementary File 1 (Tables S3 and S4). Although patients’ age did not differ between the two cohorts, a significant difference was observed between BrC patients and controls age (Cohort #1: *p* = 0.007, Cohort #2: *p* = 0.001).

### 3.2. Assessment of BrC and NBr Tissue Samples Methylation Levels

To assess cancer-specificity, promoters’ methylation levels of *APC*, *BRCA1*, *CCND2*, *FOXA1*, *PSAT1*, *RASSF1A* and *SCGB3A1* were evaluated in Cohort #1 (BrC and NBr tissue samples). Overall, BrC samples displayed higher *APC*, *CCND2*, *FOXA1*, *PSAT1*, *RASSF1A*, and *SCGB3A1* methylation levels than NrB samples (*p* < 0.001 for all genes, Supplementary File 1—Table S5), whereas no differences were found for *BRCA1*, which was not further tested (Supplementary File 1—Table S5).

**Figure 1:**
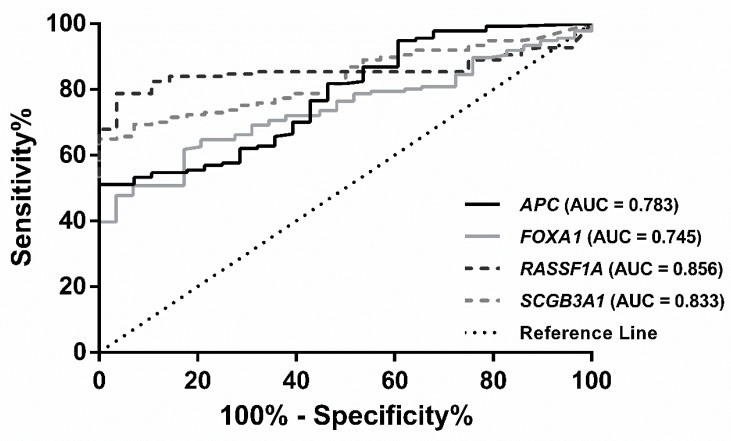
Receiver operating characteristic (ROC) curve of the four-gene panel (*APC*, *FOXA1*, *RASSF1A* and *SCGB3A1*) in Breast Cancer tissues from Cohort #1.

Subsequently, ROC curve analysis was performed, and an empirical cutoff value was determined for each gene (*APC*: 16.99, *CCND2*: 0.4171 for, *FOXA1*: 21.57, *PSAT1*: 48.05, *RASSF1A*: 114.5 and *SCGB3A1*: 67.18). All genes displayed an Area under the Curve (AUC) superior to 0.70 (Supplementary File 1—Table S6). *APC* and *SCGB3A1* disclosed 100% specificity for cancer detection, whereas *PSAT1* showed the highest sensitivity (91.97%). *RASSF1A* demonstrated the best individual performance, with 78.83% sensitivity and 96.43% specificity (Table 1). Several gene combinations were tested, and the best detection performance was achieved for the panel comprising *APC*, *FOXA1*, *RASSF1A* and *SCGB3A1*, displaying 97.81% sensitivity, 78.57% specificity and 94.50% accuracy (Table 1, Figure 1). Due to age’s difference between patients and controls, a multivariable model was constructed using logistic regression with the most informative genes and age. In this model, age did not show a significant impact in BrC detection (*p* = 0.2227). Moreover, biomarker performance was carried out restricted to BrC patient’s with similar age to controls (*p* = 0.136, Mann-Whitney for age). Similar results were obtained in biomarker performance (Supplementary File 1—Table S7).

### 3.3. Association between Promoter Methylation Levels, Molecular Subtypes and Standard Clinicopathological Parameters in Cohort #1

No significant differences in promoter methylation levels were apparent according to molecular subtype (Supplementary File 1—Figure S1), tumor grade, pathological stage or ERBB2 status in tissue samples. Nevertheless, in BrC patients, but not in controls, a significant correlation was found between *CCND2* and *RASSF1A* methylation levels and age (R = 0.194, *p* = 0.023 and R = 0.223, *p* = 0.009, respectively). Additionally, a significant association was found between histological subtype and *APC* and *SCGB3A1* methylation levels: special subtype carcinomas disclosed the lowest *SCGB3A1* methylation levels in comparison to all the other histological subtypes (*p* = 0.016) and lower *APC* methylation levels comparing with invasive lobular carcinomas (*p* = 0.0293). 

Additionally, *FOXA1* and *RASSF1A* methylation levels associated with hormone receptor status. ER+ and PR+ BrC displayed significantly lower *FOXA1* methylation levels than ER− and PR− BrC (*p* = 0.0084) or ER+ BrC (*p* = 0.0319). Contrarily, ER+ and PR+ BrC showed higher *RASSF1A* methylation levels than ER+ BrC (*p* = 0.0017). No statistical differences were observed for the remainder genes and for Cohort #2.

### 3.4. Survival Analysis

Survival analysis was only carried out for Cohort #1, due to the short-time of follow-up of Cohort #2. In the former Cohort (#1), 10 years of follow-up was considered for analysis. During this period, 37 patients (27.0%) had deceased, 24 of which were due to BrC (17.5% of all cases). At the time of the last follow-up, eight patients (5.8%) were alive with cancer and 92 patients (67.2%) were alive with no evidence of cancer.

Clinicopathological features were grouped according to: Grade (G1 & G2 vs. G3), pathological tumor size and extension (pT) stage (pT1, pT2 and pT3 & pT4), pathological regional lymph node status (pN) stage (N0 & N1 vs. N2 & N3) and stage (I, II and III & IV).

Higher tumor grade and pN stage and low *PSAT1* methylation levels categorized by P75significantly associated with worse disease-free survival (DFS) in Cox regression univariable analysis (Table 2). Nonetheless, in multivariable analysis, however, only *PSAT1* methylation levels and pN stage remained independent predictors of DFS (Table 2).

Concerning disease-specific survival (DSS), in univariable model, pN stage and grade significantly associated with prognosis. Moreover, BrC patients with high *FOXA1* promoter methylation (above P75) levels disclosed shorter DSS (Table 2). In the Cox regression multivariable model, only *FOXA1* methylation levels and pN stage retained significance for DSS prediction (Table 2).

### 3.5. Biomarker Detection Performance in ccfDNA Liquid Biopsies (Cohort #2)

**Figure 2:**
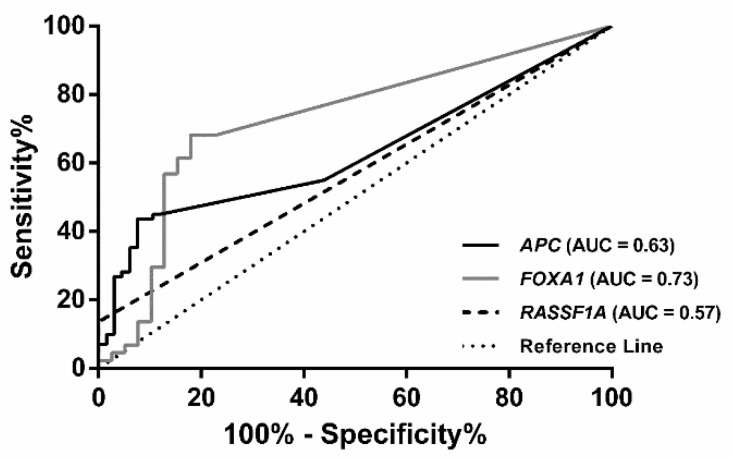
Receiver operating characteristic (ROC) curve of the three-gene panel (*APC*, *FOXA1* and *RASSF1A*) in plasma samples from breast cancer patients from Cohort #2.

The 4-gene panel (*APC*, *FOXA1*, *RASSF1A*, and *SCGB3A1*) identified in Cohort #1 was tested in ccfDNA extracted from plasma samples of Cohort #2. *APC*, *FOXA1* and *RASSF1A* promoter methylation levels significantly differed between BrC patients and AC (*p* = 0.008, *p* < 0.001 and *p* = 0.017, respectively), whereas no significant differences were found for *SCGB3A1* (*p* = 0.127) (Supplementary File 1—Table S8). Thus, *SCGB3A1* was not further analyzed. An empirical cutoff value was determined for each gene using ROC curve analysis (*APC*: 3.446, *FOXA1*: 64.38 and *RASSF1A*: 30.00). *FOXA1* disclosed the best individual performance, with 68.18% sensitivity and 82.05% specificity (Table 3). Nevertheless, the three-gene panel achieved 81.82% sensitivity and 76.92% specificity (Table 3, Figure 2). Similar to Cohort #1, a biomarker performance analysis restricted by the maximum age of the controls was performed (*p* = 0.777, Mann-Whitney for age). The biomarker performance was similar (Supplementary File 1—Table S9).

## 4. Discussion

BrC remains the major cause of cancer-related death among women worldwide. Mammographic-based screening has contributed to a 28–45% reduction in BrC mortality [4,33,34,35], disclosing 70% sensitivity, and 92% specificity for BrC detection [3]. Owing to its limitations, the need for novel detection methods, with improved accuracy and allowing for stratification of BrC aggressiveness has been emphasized [35]. In recent years, the methylome has emerged as the basis for diagnostic and prognostic biomarkers, which might be used in DNA extracted from liquid biopsies [36,37]. Considering published studies on gene promoter methylation in BrC, we aimed to define the best gene panel for detection and prognosis in tissue samples, as well as BrC detection in ccfDNA. 

From the seven most promising candidates, six (*APC*, *CCND2*, *FOXA1*, *PSAT1*, *RASSF1A* and *SCGB3A1*) confirmed its cancer-specificity, discriminating normal from cancerous tissues, although with variable performance, paralleling previous observations from our team and others [8,9,38]. Interestingly, a panel combining *APC*, *FOXA1*, *RASSF1A* and *SCGB3A1* disclosed the highest accuracy for BrC detection (94%). *APC*, *RASSF1A* and *SCGB3A1* promoter methylation have been previously tested in a diagnostic setting of fine-needle aspiration biopsy samples [8,9], whereas *FOXA1* expression has been associated with BrC subtype and prognosis [39,40], but not diagnosis. This result compares well with other gene promoter methylation panels that have been reported, disclosing 60–80% sensitivity and 78–100% specificity, and differences in performance are most likely related to biological sample type (tissue vs. bodily fluids) and methylation assessment methods [41]. 

Since a major goal of this study was to define a panel for BrC detection, ideally its performance should be homogenous regardless of molecular subtype. Thus, we used IHC for tumor subtyping, although acknowledging its limitations in triple negative breast cancer (TNBC)/basal-type classification and luminal A vs. luminal B discrimination [42,43,44]. Interestingly, no association was found between gene promoter methylation and BrC molecular subtype, suggesting that the gene panel might be effective across molecular subtypes. Some studies have associated DNA methylation and specific molecular subtypes, but these have used a similar proportion of all subtypes or have only analyzed a specific subtype [13,45,46], or even used different methods [10,11,47,48]. Our results, however, are based on a consecutive series of cases, which were not selected according to subtype, and, thus, ERBB2-like and TNBC tumor subtypes are, naturally, in a smaller proportion, which might limit statistical analysis. Nevertheless, *APC* and *SCGB3A1* promoter methylation levels associated with specific histological subtypes, confirming previous observations [49]. Interestingly, *FOXA1* and *RASSF1A* promoter methylation levels associated with hormone receptor status. Although the reason for these associations is unclear, similar results for *RASSF1A* have been reported [50,51]. On the other hand, the higher *FOXA1* promoter methylation observed in hormone receptor negative BrC is in accordance with reported *FOXA1* hypermethylation in basal BrC cell lines [29,52].

Because liquid biopsies represent a promising method for minimally-invasive early cancer detection [24,28], we tested the selected gene panel in ccfDNA. Interestingly, three genes retained diagnostic significance (*APC*, *FOXA1* and *RASSF1A*), whereas *SCGB3A1* did not discriminate BrC patients from controls. These results are in accordance with another study [53], and might be due to differences in sample number and methylation assessment methods [54]. Moreover, the frequency of gene methylation in Cohort #2 was similar to that previous reported in ccfDNA (Table 4) [25,54,55,56,57,58,59,60,61,62,63].

Nonetheless, the three gene-panel identified BrC with sensitivity, specificity and accuracy higher than 75%. Thus, this panel disclosed a better combination of sensitivity and specificity than most published studies using plasma or serum samples (Table 5), excepting those of Skvortosova et al. (three-gene panel in plasma) and Kim et al. (four-gene panel in serum) [25,54,55,56,57,58,59,60,61,62,63]. Nevertheless, the same authors tested a very limited set of samples (20 BrC, 15 fibroadenomas and 10 healthy donors). Importantly, we used a 4-color multiplex assay that, when compared with the most widely reported two-color multiplex assays represents a faster method and requires less amounts of DNA, thus facilitating its use in clinical routine [36,37,64,65,66]. Hence, this gene-panel may constitute an appealing alternative to conventional diagnostic methods, due to its less-invasive characteristics and to also detect also women with a dense breast.

Although BrC displays high mortality and recurrence rate, clinical course is heterogeneous and perfecting disease prognostication might improve patient management. Interestingly, lower *PSAT1* promoter methylation independently predicted for worse DFS. The potential of *PSAT1* methylation to predict BrC recurrence has been previously reported in early diagnosed luminal-type BrC. Futhermore, a correlation between high *PSAT1* methylation levels, on the one hand, and low *PSAT1* mRNAs levels and better outcome, on the other, were disclosed [14]. Interestingly, high *PSAT1* expression were associated with poor outcome in nasopharyngeal carcinoma [67]. These data are in accordance with our findings. Furthermore, high *FOXA1* methylation levels independently predicted shorter DSS, a finding that, to best of our knowledge, has not been reported, thus far. Remarkably, *FOXA1* expression was previously shown to associate with good prognosis and response to endocrine therapy in BrC patients [39,40], and, thus, promoter methylation is the most likely mechanism underlying *FOXA1* downregulation in BrC. In Cohort #1, *RASSF1A* methylation levels did not show prognostic value, which is in accordance with some previous publications [68,69,70]. Nonetheless, other studies have found *RASSF1A* hypermethylation as a poor prognosis marker in BrC, associating with shorter DFS and DSS [9,22,71]. This discrepancy might be due to differences in sample type and methodologies. Because a meta-analysis suggested that *RASSF1A* methylation is, indeed, associated with worse DFS and DSS [72], additional studies are needed to definitively establish the prognostic value of *RASSF1A* promoter methylation in BrC.

The retrospective nature of Cohort #1, the limited sample size of Cohort #2 and the age differences between BrC patients and controls in both series constitute the main limitations of our study. Nonetheless, it should be emphasized that the use of a multiplex assay for a three-gene panel that is able to accurately detect BrC in ccfDNA, regardless of tumor subtype, constitutes a step forward in this field and allow for a swifter translation into routine clinical practice. Indeed, owing to its characteristics, this panel might not only be useful for BrC detection, but also for disease monitoring which deserves further exploration.

## Figures and Tables

**Table 1:** Performance of promoter gene methylation as biomarkers for detection of breast cancer (Brc) in tissue samples.

Genes	Sensitivity %	Specificity %	PPV ^a^ %	NPV ^b^ %	Accuracy %
*APC*	51.09	100.0	100.0	29.47	59.39
*CCND2*	72.26	92.86	98.02	40.63	75.76
*FOXA1*	62.04	82.14	94.44	30.67	65.45
*PSAT1*	91.24	50.00	89.93	53.85	84.24
*RASSF1A*	78.83	96.43	99.08	48.21	81.82
*SCGB3A1*	64.96	100.0	100.0	36.84	70.91
*APC/FOXA1* *RASSF1A/SCGB3A1*	97.81	78.57	95.71	88.00	94.55

^a^ PPV—Positive Predictive Value; ^b^ NPV—Negative Predictive Value.

**Table 2:** Cox regression models assessing the potential of clinical and epigenetic variables in the prediction of disease-free survival for 122 patients with BrC and disease-specific survival for 127 patients with BrC.

Disease-Free Survival	Variable	HR ^a^	CI ^b^ (95%)	*p* Value
Univariable	Grade			
	G1	1		
	G2 ^c^ & G3	2.054	1.029–4.098	0.041
	pN Stage			
	N0 ^d^ & N1	1		
	N2 & N3	3.894	1.940–7.812	<0.001
	*PSAT1*			
	>P75 ^e^	1		
	≤P75	3.707	1.133–12.127	0.030
Multivariable	Grade			
	G1	1		
	G2 & G3	1.490	0.717–3.096	0.286
	pN Stage			
	N0 & N1	1		
	N2 & N3	4.345	2.114–8.930	<0.001
	*PSAT1*			
	>P75 ^e^	1		
	≤P75	3.613	1.077–12.123	0.038
**Disease-Specific Survival**	**Variable**	**HR ^a^**	**CI ^b^ (95%)**	***p*** **Value**
Univariable	Grade			
	G1	1		
	G2 & G3	2.725	1.155–6.428	0.022
	pN Stage			
	N0 & N1	1		
	N2 & N3	4.061	1.814–9.089	0.001
	*FOXA1*			
	≤P75 ^f^	1		
	>P75	2.678	1.200–5.978	0.016
Multivariable	Grade			
	G1	1		
	G2 & G3	2.005	0.082–4.866	0.124
	pN Stage			
	N0 & N1	1		
	N2 & N3	4.855	1.981–10.611	<0.001
	*FOXA1*			
	≤P75 ^f^	1		
	>P75	2.710	1.161–6.324	0.021

^a^ HR—Hazard Ratio; ^b^ CI—Confidence Interval; ^c^ G—Grade; ^d^ N—Regional lymph node status; ^e^ P75—Percentile 75 of methylation levels of *PSAT1*; ^f^ P75—Percentile 75 of methylation levels of *FOXA1*.

**Table 3:** Performance of promoter gene methylation as biomarkers for detection of BrC in plasmas samples.

Genes	Sensitivity %	Specificity %	PPV ^a^ %	NPV ^b^ %	Accuracy %
*APC*	27.27	94.87	85.71	53.62	59.04
*FOXA1*	68.18	82.05	81.08	69.57	74.70
*RASSF1A*	13.64	100.0	100.0	50.65	54.22
*APC/FOXA1/RASSF1A*	81.82	76.92	80.00	78.95	79.52

^a^ PPV—Positive Predictive Value; ^b^ NPV—Negative Predictive Value.

**Table 4:** Frequency of positive cases [*n*(%)] for methylation levels of cancer-related genes in ccfDNA.

Genes/Panel	Controls (Healthy Donors)		Patients		References
	*n*	%	*n*	%	
*HIC-1/RARβ2/RASSF1A* ^a^	0/10	0%	18/20	90%	[60]
*APC*	0/38	0%	8/47	17%	[55]
*GSTP1*	0/38	0%	12/47	26%	
*RARβ2*	3/38	8%	12/47	26%	
*RASSF1A*	2/38	5%	15/47	32%	
*APC/GSTP1/RARβ2/RASSF1A*	5/38	13%	29/47	62%	
*ATM*	0/14	0%	13/50	26%	[58]
*RASSF1A*	0/14	0%	7/50	14%	
*ATM/RASSF1A*	0/14	0%	18/50	36%	
*RARβ2*	8/125	6%	103/119	87%	[54]
*RASSF1A*	6/125	5%	39/119	33%	
*SCGB3A1*	0/125	0%	36/119	30%	
*Twist*	10/125	8%	65/119	55%	
*RARβ2/RASSF1A/SCGB3A1/Twist*	23/125	18%	117/119	98%	
*ITIH5*	7/135	6%	19/138	14%	[25]
*DKK3*	2/135	2%	41/138	30%	
*RASSF1A*	25/135	26%	64/138	47%	
*ITIH5/DKK3/RASSF1A*	42/135	31%	92/138	67%	
*CDH1*	0/25	0%	24/50	48%	[57]
*RASSF1A*	0/25	0%	32/50	64%	
*CDH1/RASSF1A*	0/25	0%	38/50	76%	
*SFN*	143/245	58%	197/268	74%	[59]
*P16*	41/245	17%	60/268	33%	
*hMLH1*	35/245	14%	75/268	28%	
*HOXD13*	6/245	2%	37/268	14%	
*PCDHGB7*	116/245	48%	149/268	56%	
*RASSF1A*	25/245	10%	46/248	17%	
*SFN/P16/hMLH1/HOXD13/PCDHGB7/RASSF1A* ^b^	68/245	28%	213/268	80%	
*ESR1*	35/74	47%	80/106	75%	[56]
*14-3-3-σ*	28/74	38%	69/106	65%	
*ESR1/14-3-3-σ* ^b^	33/74	45%	86/106	81%	
*GSTP1*	2/87	2%	4/101	4%	[62]
*RARβ2*	0/87	0%	7/101	7%	
*RASSF1A*	4/87	5%	12/101	12%	
*GSTP1/RARβ2/RASSF1A*	6/87	7%	22/101	22%	
*DAPK1*	0/12	0%	23/26	88%	[63]
*RASSF1A*	1/12	8%	18/26	69%	
*DAPK1/RASSF1A*	1/12	8%	25/26	96%	
*APC*	1/19	5%	23/79	30%	[61]
*ESR1*	2/19	11%	16/79	20%	
*RASSF1A*	0/19	0%	28/79	35%	
*APC/ESR1/RASSF1A*	3/19	16%	42/79	53%	
*APC*	2/39	5%	12/44	27%	---
*FOXA1*	7/39	18%	30/44	68%	
*RASSF1A*	0/39	0%	6/44	14%	
*APC/FOXA1/RASSF1A*	9/39	23%	36/44	82%	

^a^ No information about single gene methylation; ^b^ The cut-off used in the panel was different the one used in the single gene analysis.

**Table 5:** Comparison of sensitivity and specificity of previously published panels with values obtained.

Panels	Sensitivity (%)	Specificity (%)	Specimen Type	Methods	References
*HIC-1/RARβ2/RASSF1A*	90	100	Plasma	MSP ^a^	[60]
*APC/GSTP1/RARβ2/RASSF1A*	62	87	Plasma	QMSP ^b^	[55]
*ATM/RASSF1A*	36	100	Plasma	QMSP ^b^	[58]
*RARβ2/RASSF1A/SCGB3A1/* *Twist*	98.3	81.6	Serum	Two-steps QMSP ^b^	[54]
*ITIH5/DKK3/RASSF1A*	67	72	Serum	QMSP ^b^	[25]
*CDH1/RASSF1A*	76	90	Serum	MSP ^a^	[57]
*SFN/P16/hMLH1/HOXD13/PCDHGB7/RASSF1A*	79.6	72.4	Serum	QMSP ^b^	[59]
*ESR1/14-3-3-σ*	81	55	Serum	QMSP ^b^	[56]
*GSTP1/RARβ2/RASSF1A*	22	93	Serum	One-step MSP ^a^	[62]
*DAPK1/RASSF1A*	96	71	Serum	MSP ^a^	[63]
*APC/ESR1/RASSF1A*	53	84	Serum	QMSP ^b^	[61]
*APC/FOXA1/RASSF1A*	81.82	76.92	Plasma	Multiplex QMSP ^b^	---

^a^ MSP—Methylation-Specific Polimerase Chain Reaction; ^b^ QMSP—Quantitative Methylation-Specific Polimerase Chain Reaction.

## Supplementary Materials

The following are available online at http://www.mdpi.com/2077-0383/7/11/420/s1, Supplementary File 1 which includes: Table S1: Primers sequences and QMSP conditions for each gene studied in tissues samples; Table S2: Primers and probe sequences for each gene studied in plasma samples; Table S3: Clinical—pathological data of normal breast tissue, and (NBr) breast cancer (BrC) patient’s (Cohort #1); Table S4: Clinical and pathological data of asymptomatic controls (AC) and Breast Cancer (BrC) patients (Cohort #2); Table S5: Frequency of positive cases [*n*(%)] and distribution of methylation levels of cancer-related genes in tissues from Cohort #1 [gene/ACTB × 1000 median (Interquartile range (IQR))]; Table S6: Area Under the Curve (AUC) of Receiver Operating Characteristic (ROC) Curve for each gene in tissues from Cohort#1; Table S7: Performance of promoter gene methylation as biomarkers for detection of BrC in tissue samples from patients with age below 70 years (controls *n* = 27 and tumors *n* = 108); Table S8: Frequency of positive cases [*n*(%)] for methylation levels of cancer-related genes in ccfDNA of Cohort #2.; Table S9: Performance of promoter gene methylation as biomarkers for detection of BrC in plasmas samples from patients with age below 66 years (controls *n* = 39 and tumors *n* = 25); Figure S1: Boxplots of *APC* (a), *BRCA1* (b), *CCND2* (c), *FOXA1* (d), *PSAT1* (e), *RASSF1A* (f) and *SCGB3A1* (g) methylation levels in the breast cancer molecular subtypes and normal breast tissues.
