# Supplementary material for: A DNA Methylation-Based Test for Breast Cancer Detection in Circulating Cell-Free DNA
Source: J Clin Med. 2018 Nov 7;7(11):420. doi: 10.3390/jcm7110420 (PMC6262630; doi:10.3390/jcm7110420)

Salta & Nunes et al.-Supplementary File

Table S1– Primers sequences and QMSP conditions for each gene studied in tissues samples.
Table S2 – Primers and probe sequences for each gene studied in plasma samples.
Table S3 – Clinical—pathological data of normal breast tissue, and (NBr) breast cancer (BrC) patient's (Cohort#1).
Table S4 – Clinical and pathological data of asymptomatic controls (AC) and BrC patients (Cohort#2).
Table S5 – Frequency of positive cases [n(%)] and distribution of methylation levels of cancer-related genes in tissues from Cohort#1 [gene/ACTB x1000 median ((Interquartile range)IQR)].
Table S6 – Area Under the Curve (AUC) of Receiver Operating Characteristic (ROC) Curve for each gene in tissues from Cohort#1.
Table S7 – Performance of promoter gene methylation as biomarkers for detection of BrC in tissue samples from patients with age below 70 years (controls n=27 and tumors n=108).
Table S8 – Frequency of positive cases [n(%)] for methylation levels of cancer-related genes in ccfDNA of Cohort#2.
Table S9 – Performance of promoter gene methylation as biomarkers for detection of BrC in plasmas samples from patients with age below 66 years (controls n=39 and tumors n=25)

Supplementary Figures Legends:
Figure S1 – Boxplots of *APC* (a), *BRCA1* (b), *CCND2* (c), *FOXA1* (d), *PSAT1* (e), *RASSF1A* (f) and *SCGB3A1* (g) methylation levels in the breast cancer molecular subtypes and normal breast tissues.

Supplementary Tables

Table S1 – Primers sequences and QMSP conditions for each gene studied in tissues samples.

Gene	Primers	Annealing Temperature ºC
ACTβ	F – 5′ TGG TGA TGG AGG AGG TTT AGT AAG T 3′	60ºC
	R – 5′ ACC AAT AAA ACC TAC TCC TCC CTT AA 3′	
APC	F – 5′ TGT GTT TTA TTG CGG AGT GC 3′	62ºC
	R – 5′ CAC ATA TCG ATC ACG TAC GC 3′	
BRCA1	F – 5′ TTT CGT GGT AAC GGA AAA GC 3′	60ºC
	R – 5′ ATC TCA ACG AAC TCA CGC CG 3′	
CCND2	F – 5′ TTT GAT TTA AGT ATG CGT TAG AGT ACG 3′	62ºC
	R – 5′ ACT TTC TCC CTA AAA ACC GAC TAC G 3′	
FOXA1	F – 5′ CGA CGT TAA GAC GTT TAA GC 3′	62ºC
	R – 5′ CGC TCA ACG TAA ACA TCT TAC 3′	
PSAT1	F – 5′ TGG GTT TGG TTT CGT TAA GTT GT 3′	64ºC
	R – 5′ ACG TAC TCC CGC CTA AAC CTC 3′	
RASSF1A	F – 5′ AGC GAA GTA CGG GTT TAA TC 3′	60ºC
	R – 5′ ACA CGC TCC AACC GA ATA 3′	
SCGB3A1	F – 5′ GTA CGG TCG TGA GCG GAG C 3′	64ºC
	R – 5′ GAA ACT TCT TAT ACC CGA TCC TC 3′	

Table S2 – Primers and probe sequences for each gene studied in plasma samples.

Gene		Sequences
ACTβ	Primers	F – 5′ TGG TGA TGG AGG AGG TTT AGT AAG T 3′
		R – 5′ ACC AAT AAA ACC TAC TCC TCC CTT AA 3′
	Probe	5′ Cy5 – ACC ACC ACC CAA CAC ACA ATA ACA AAC ACA – QSY 3′
APC	Primers	F – 5′ TGT GTT TTA TTG CGG AGT GC 3′
		R – 5′ CAC ATA TCG ATC ACG TAC GC 3′
	Probe	5′VIC – CAATCGACGAACTCCCGAC – MGB 3′
FOXA1	Primers	F – 5′ CGA CGT TAA GAC GTT TAA GC 3′
		R – 5′ CGC TCA ACG TAA ACA TCT TAC 3′
	Probe	5′ FAM -ATA TAC GAA TAA AAC GAC TTA ACG – MGB 3′
RASSF1A	Primers	F – 5′ AGC GAA GTA CGG GTT TAA TC 3′
		R – 5′ ACA CGC TCC AACC GA ATA 3′
	Probe	5′ NED – CGG GAG TTG GTA TTC GTT GGG CG – QSY3′
SCGB3A1	Primers	F – 5′ GTA CGG TCG TGA GCG GAG C 3′
		R – 5′ GAA ACT TCT TAT ACC CGA TCC TC 3′
	Probe	5′ FAM – GCC GAC CTC GCC CGC GCT CCT AAA – Iowa Black RQ-Sp 3′

Table S3 – Clinical—pathological data of normal breast tissue, and (NBr) breast cancer (BrC) patient's (Cohort#1)

Clinicopathologic features	BrC	NBr
Patients (no.)	137	28
Age median (range)	62 (33-88)	54 (30-70)
Molecular subtype (no).		
Luminal A-like	29	n.a.[a]
Luminal B-like	74	
ERBB2 overexpression-like	12	
TNBC[b]	22	
Histological Type		
Invasive Ductal Carcinoma	116	n.a.
Invasive Lobular Carcinoma	8	
Special Subtype Carcinomas	5	
Mixed Type Carcinoma	8	
Grade (no.)		
G1	13	n.a.
G2	63	
G3	56	
n.a.	5	
Estrogen Receptor Status		
Positive	102	n.a.
Negative	35	
Progesterone Receptor Status		
Positive	66	n.a.
Negative	71	
ERBB2 Receptor Status		
Positive	25	n.a.
Negative	112	
Pathological T Stage (no.)		
pT1	42	n.a.
pT2	84	
pT3	7	
pT4	2	
pTx	2	
Pathological N Stage (no.)		
pN0	52	n.a.
pN1	45	
pN2	17	
pN3	19	
pNx	4	
Stage (no.)		
I	21	n.a.
II	72	
III & IV	43	

n.a. – not applicable; TNBC – Triple Negative Breast Cancer; ERBB2, Erb-b2 receptor tyrosine kinase.

Table S4 – Clinical and pathological data of asymptomatic controls (AC) and BrC patients (Cohort#2).

Clinicopathologic features	BrC	AC
Patients (n)	44	39
Age median (range)	63 (37-91)	52 (46-65)
Molecular subtype (no).		
Luminal	41	n.a.[a]
TNBC[b]	2	
Histological Type		
Invasive Ductal Carcinoma	33	n.a.
Others	11	
Grade (no.)		
G1	6	n.a.
G2	18	
G3	19	
Not determined	1	
Estrogen Receptor Status		
Positive	40	n.a.
Negative	3	
Not determined	1	
Progesterone Receptor Status		
Positive	37	n.a.
Negative	6	
Not determined	1	
ERBB2 Receptor Status		
Positive	3	n.a.
Negative	40	
Not determined	1	
Pathological Stage (no.)		
I	16	n.a.
II	19	
III	9	
Not determined	1	

[a] n.a. – not applicable; [b] TNBC – Triple Negative Breast Cancer; [c] Breast cancer diagnosis by cytology, patient refused treatment.

Table S5 – Frequency of positive cases [n(%)] and distribution of methylation levels of cancer-related genes in tissues from Cohort#1 [gene/ACTB x1000 median (IQR)]

Genes	NBr		BrC		*p* value
	n (%)	Median (IQR[a])	n (%)	Median (IQR)	
APC	0/28 (0%)	1.172 (0.4732-3.875)	70/137 (51%)	24.10 (1.429-233.8)	<0.0001
BRCA1	21/28 (75.0%)	0.0830 (0.0043-0.2785)	135/137 (98.5%)	0.1376 (0.0519-0.4138)	0.111
CCND2	2/28 (7.1%)	0.0285 (0.0001-0.2944)	99/137 (72.3%)	5.552 (0.3338-31.29)	<0.0001
FOXA1	5/28 (17.9%)	12.25 (7.725-19.00)	85/137 (62.0%)	28.33 (13.51-61.73)	<0.0001
PSAT1	14/28 (50.0%)	66.83 (25.33-248.7)	125/137 (91.2%)	222.15 (103.7-351.5)	<0.0001
RASSF1A	1/28 (3.6%)	13.90 (2.277-31.47)	108/137 (78.8%)	329.84 (141.4-560.7)	<0.0001
SCGB3A1	0/28 (0%)	1.218 (0.2794-5.715)	89/137 (65.0%)	217.27 (3.225-577.8)	<0.0001

[a] IQR – Interquartile range; *p* value obtained from Mann-Whitney test.

Table S6 – Area Under the Curve (AUC) of Receiver Operating Characteristic (ROC) Curve for each gene in tissues from Cohort#1.

Genes	AUC
APC	0.783
CCDN2	0.852
FOXA1	0.745
PSAT1	0.713
RASSF1A	0.856
SCGB3A1	0.833

Table S7 – Performance of promoter gene methylation as biomarkers for detection of BrC in tissue samples from patients with age below 70 years (controls n=27 and tumors n=108)

Genes	Sensitivity %	Specificity %	PPV[a] %	NPV[b] %	Accuracy %
APC	50.9	100.0	100.0	33.8	60.7
CCND2	71.3	92.6	97.5	44.6	75.6
FOXA1	63.0	81.5	93.2	35.5	66.7
PSAT1	90.7	51.9	88.3	58.3	83.0
RASSF1A	75.0	96.3	98.8	49.1	79.3
SCGB3A1	63.9	100.0	100.0	40.9	71.1
APC/FOXA1 RASSF1A/ SCGB3A1	98.15	77.78	94.64	91.30	94.7

[a] PPV – Positive Predictive Value; [b] NPV – Negative Predictive Value;

Table S8 – Frequency of positive cases [n(%)] for methylation levels of cancer-related genes in ccfDNA of Cohort#2

Genes	AC		BrC		*p* value
	n	%	n	%	
APC	2/39	5.13	12/44	27.27	0.008
FOXA1	7/39	17.95	30/44	68.18	< 0.001
RASSF1A	0/39	0	6/44	13.64	0.017
SCGB3A1	5/39	12.82	12/44	27.27	0.127

p value obtained from Mann-Whitney test.

Table S9 – Performance of promoter gene methylation as biomarkers for detection of BrC in plasmas samples from patients with age below 66 years (controls n=39 and tumors n=25)

Genes	Sensitivity %	Specificity %	PPV[a] %	NPV[b] %	Accuracy %
APC	28.00	94.87	77.78	67.27	68.75
FOXA1	64.00	82.05	69.57	78.05	75.00
RASSF1A	4.000	100.0	100.0	61.91	62.50
APC/FOXA1/RASSF1A	76	76.92	67.86	83.33	76.56

[a] PPV – Positive Predictive Value; [b] NPV – Negative Predictive Value

Figure S1. Boxplots of APC (a), BRCA1 (b), CCND2 (c), FOXA1 (d), PSAT1 (e), RASSF1A (f) and SCGB3A1 (g) methylation levels in the breast cancer molecular subtypes and normal breast tissues.

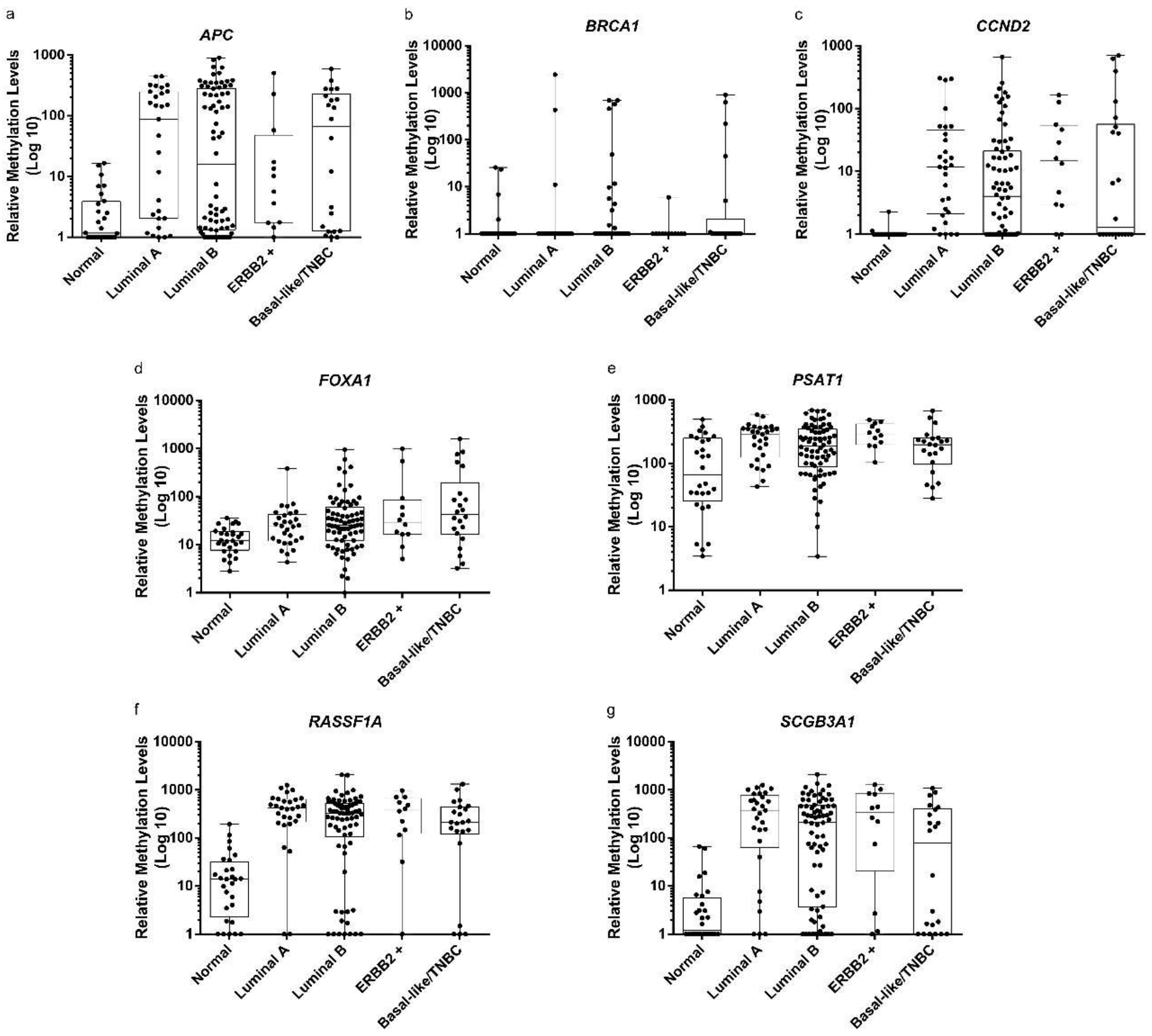

Supplement: Supplementary file 1 [file jcm-07-00420-s001.zip › jcm-369542-SI.pdf]
